# Protective Effect of Curcumin against the Liver Toxicity Caused by Propanil in Rats

**DOI:** 10.1155/2014/853697

**Published:** 2014-10-29

**Authors:** Chiagoziem A. Otuechere, Sunny O. Abarikwu, Victoria I. Olateju, Azeezat L. Animashaun, Oluwafemi E. Kale

**Affiliations:** ^1^Department of Chemical Sciences, Redeemer's University, PMB 2011, Mowe, Ogun, Nigeria; ^2^Department of Pharmacology, College of Medicine, University of Lagos, PMB 12003, Lagos, Nigeria

## Abstract

We investigated the protective effects of curcumin on propanil-induced alterations in biochemical indices in blood and liver of male Wistar rats. The study consisted of four treatment groups, with six animals each, designated as control, propanil (20mg/kg), curcumin(50 mg/kg), and curcumin (50 mg/kg) + propanil (20 mg/kg). Rats were administered their respective doses orally, every other day, for 28 days. Propanil administration elicited significant (*P* < 0.001) increases in plasma aspartate aminotransferase and alkaline phosphatase activities, by 24% and 56%, respectively, compared to the control. Treatment with propanil elevated bilirubin, creatinine, and total cholesterol levels in rats, but these were not significant relative to controls. Administration of propanil to rats significantly (*P* < 0.001) increased lipid peroxidation levels. However, catalase activity, vitamin C, and reduced glutathione levels were significantly reduced. Exposure to propanil did not produce any significant changes in packed cell volume, neutrophils, and leukocyte counts. The supplementation of curcumin attenuated the adverse effects of propanil intoxication by reducing lipid peroxidation levels and restored the levels of serum enzymes and reduced glutathione. The present study showed that propanil increased oxidative stress and altered some biochemical parameters in the rats but curcumin could afford some protection to attenuate propanil-induced toxicity in the liver.

## 1. Introduction

Curcumin (diferuloylmethane) is a yellow colouring ingredient of the spice turmeric obtained from the rhizome of* Curcuma longa* Linn (Zingiberaceae). It is a perennial herb distributed mainly throughout tropical and subtropical regions of the world [1]. Curcumin possesses anti-inflammatory, immunomodulatory, and antiatherogenic activities and is a potent inhibitor of various reactive oxygen-generating enzymes [2, 3]. It has been used in indigenous herbal medicine for the treatment of inflammatory and liver disorders. Antioxidative properties of curcumin are well documented. Curcumin is a potent scavenger of reactive oxygen species including superoxide anion radicals and hydroxyl radicals. It has also been reported to inhibit erythrocyte lipid peroxidation [4]. Curcumin administration attenuated the arsenic, gentamicin, and acetaminophen-induced oxidative stress in rats [5–7]. Curcumin also prevented free radical formation-induced myocardial ischemia and paraquat-induced lung injury in rats [8, 9]. Additionally, curcumin protected against diazinon-induced toxicity in blood, liver, and erythrocyte of male Wistar rats [10]. Furthermore, Canales-Aguirre and coworkers [11] had also reported the protective effects of curcumin against the oxidative damage in the hippocampus of rats after exposure to parathion.

Propanil (3, 4-dichloropropioanilide) is an acylanilide herbicide used to control barnyard grass, broadleaf weeds, and for the postemergent treatment of rice [12]. The widespread use of the herbicide on rice and wheat crops means that humans could be at risk of high level exposure. This necessitated the World Health Organization to recognize propanil as slightly hazardous in terms of human risk [13]. However, in a retrospective study assessing the clinical features of sixteen patients admitted for acute propanil poisoning, the authors advocated that the mild-to-moderate toxicity label of propanil should be revisited by the WHO [14]. Earlier reports by McMillan and coworkers [15] indicated that exposure to propanil had been associated with toxicity in humans and other mammals. Furthermore, it had been demonstrated that propanil could potentially induce cytotoxicity and nephrotoxicity* in vitro* [16]. A recent study reported that propanil induced dose-dependent histopathological changes in the liver and kidney tissues of exposed mice [17].

Antioxidants had been proven to play an important role in the regulation of a vast array of physiological and pathological processes. They principally contribute to the protection of cells and tissues against deleterious effects of reactive oxygen species and other free radicals.

The aim of this study was to determine the effect of subacute propanil exposure in the plasma of male rats and to assess whether these effects could be ameliorated by cotreatment with curcumin. To achieve this aim, rats were given propanil and curcumin by oral gavage for 28 days, after which malondialdehyde (MDA) and GSH levels and GST and CAT activities as well as other biochemical endpoints were evaluated.

## 2. Materials and Methods

### 2.1. Chemicals

Commercial herbicide, propanil, was purchased from Harvest Field Industries Limited, Lagos State, Nigeria. Curcumin was obtained from Sigma Chemicals Corp, St. Louis, MO, USA. Other reagents were of analytical grade and the purest quality available.

### 2.2. Animals and Treatments

Albino rats, weighing between 150 g and 200 g, purchased from covenant farm animal house located at Ibadan, Oyo State, Nigeria, were used in this study. The animals were kept in well-ventilated cages at room temperature and under controlled conditions of ambient temperature (25°C) at the Redeemer's University Animal House Facility, Mowe, Ogun State, Nigeria. They were maintained on normal laboratory chow and water* ad libitum.* The experiment was approved by the Animal Ethics Committee of the Redeemer's University.

### 2.3. Animal Treatment and Sample Collection

Animals were divided into 4 experimental groups (*n* = 6). The first group received olive oil at a dose of 2 mL/kg body weight. The second group received an oral administration of propanil at a dose of 20 mg/kg/bodyweight equivalent to one-fifth of the oral dose used previously in our laboratories [18]. The third group received an oral administration of curcumin at a dose of 50 mg/kg/bodyweight. This dose was selected on the basis of a previous study [19]. The fourth group received both curcumin (50 mg/kg/bodyweight) + propanil (20 mg/kg/body weight). Curcumin and propanil were dissolved in olive oil at a dose of 2 mg/kg bw. The treatments (using intragastric feeding needle) were three times a week for a total of 28 days. Rats were sacrificed, after the last dose of administration and an overnight fast, by cervical dislocation and blood was obtained by cardiac puncture. Ethylenediaminetetraacetic acid was used as an anticoagulant. Plasma was obtained by centrifugation of samples at 4000 rpm for 5 minutes and stored at −20°C till analysis. Liver of rats was excised, homogenized in phosphate buffer (0.1 M, pH 7.4), and centrifuged at 4,500 rpm and the supernatants were used for the various biochemical measurements.

### 2.4. Biochemical Assays

Alkaline phosphatase (ALP), aspartate aminotransferase (AST), alanine aminotransferase (ALT), total cholesterol, creatinine, and bilirubin were estimated in plasma using Randox commercial kits. Lipid peroxidation (LPO) was determined by measuring the formation of thiobarbituric acid reactive substances (TBARS) according to the method of Varshney and Kale [20] while glutathione S-transferase (GST) activity was determined according to Habig et al. [21]. Catalase activity was determined according to the method of Sinha [22]. Vitamin E was estimated by the method of Desai [23]. Briefly, 0.3 mL of ethanol and 0.4 mL diethyl ether were added to 1 mL plasma and centrifuged for 5 minutes at 4000 rpm. The ascorbic acid (Vitamin C) present in the liver tissue was determined by the spectrophotometric method of Omaye et al. [24]. The method of Beutler et al. [25] was followed in estimating the level of glutathione (GSH). This method is based upon the development of a relatively stable (yellow) color when 5′, 5′-dithiobis-(2-nitrobenzoic acid) (Ellman's reagent) is added to sulfhydryl compounds. The chromophoric product resulting from the reaction of Ellman's reagent with the reduced glutathione, 2-nitro-5-thiobenzoic acid had a molar absorption at 412 nm.

### 2.5. Hematological Analyses

Noncoagulated blood was tested, after collection, for packed cell volume (PCV) and differential white blood cells count according to the procedure outlined by Cheesbrough and McArthur [26]. Haematocrit centrifuge, reader, and capillary tubes were used to determine the PCV. A blood smear was prepared by dragging the blood over the slide with the help of another slide. The smear was then diluted with Leishman's stain and allowed to dry. The stained smears were microscopically observed under oil immersion to determine the number of each cell type per cubic millimeter of blood. The stain used allowed exact identification of neutrophils and leukocytes. Protein level was estimated according to the method described by Lowry et al. [27], using bovine serum albumin as standard.

### 2.6. Statistical Analysis

All values were expressed as mean ± standard deviation (SD) of six observations. Differences between the groups were determined by one-way analysis of variance (ANOVA) and post hoc testing was performed for intergroup comparisons using Tukey's test using Graph Pad Prism 3 software. Values were regarded as significantly different at *P* < 0.001.

## 3. Results

### 3.1. Effect of Curcumin on Plasma Biochemical Parameters in Propanil-Treated Rats

**Figure 1:**
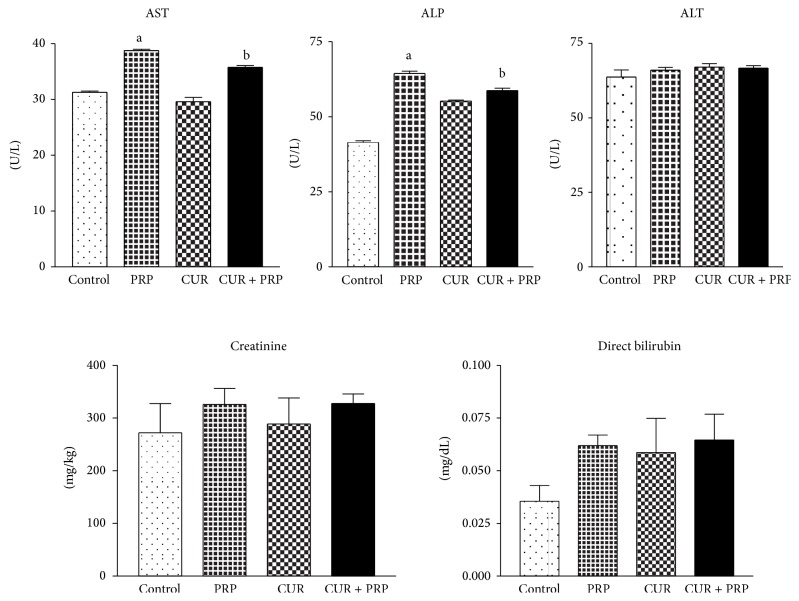
Effect of curcumin (CUR) on some plasma biochemical indices in rats orally treated with propanil. Values are expressed as mean ± SD for six animals per group. ^a^ *P* < 0.001 compared to control group. ^b^ *P* < 0.001 compared to propanil group.

Evaluation of plasma marker indices in rats exposed to propanil (20 mg/kg/bodyweight) showed that there were significant (*P* < 0.001) increases in AST and ALP activities by 24% and 56%, respectively, when compared with controls (Figure 1). The result presented in Figure 1 also showed insignificant differences in the activity of ALT and total bilirubin in propanil-challenged rats when compared with the controls. Curcumin, administered alone, had similar effects on plasma biochemical parameters when compared with controls. However, treatment with both curcumin and propanil resulted in a significant reduction in AST and ALP activities.

### 3.2. Effect of Curcumin on Some Hematological Parameters and Cholesterol Levels in Propanil-Treated Rats

The hematological properties of rats exposed to propanil are shown in Table 1. Treatment with propanil alone did not significantly decrease PCV and neutrophil levels relative to the control. Treatment with propanil elevated the total leukocyte counts but this was not significant relative to the control. However, the curcumin + propanil group showed a significant (*P* < 0.05) increase in the total leukocyte counts when compared to the control. Furthermore, there were no significant differences in the level of total cholesterol in propanil-treated rats when compared with the control. Although the curcumin + propanil group elicited a significant increase in the cholesterol levels when compared with control animals, however, there was no significant change when compared with the propanil-treated group (Table 1).

### 3.3. Effect of Curcumin on the Antioxidant Status of Propanil-Treated Rats

**Figure 2:**
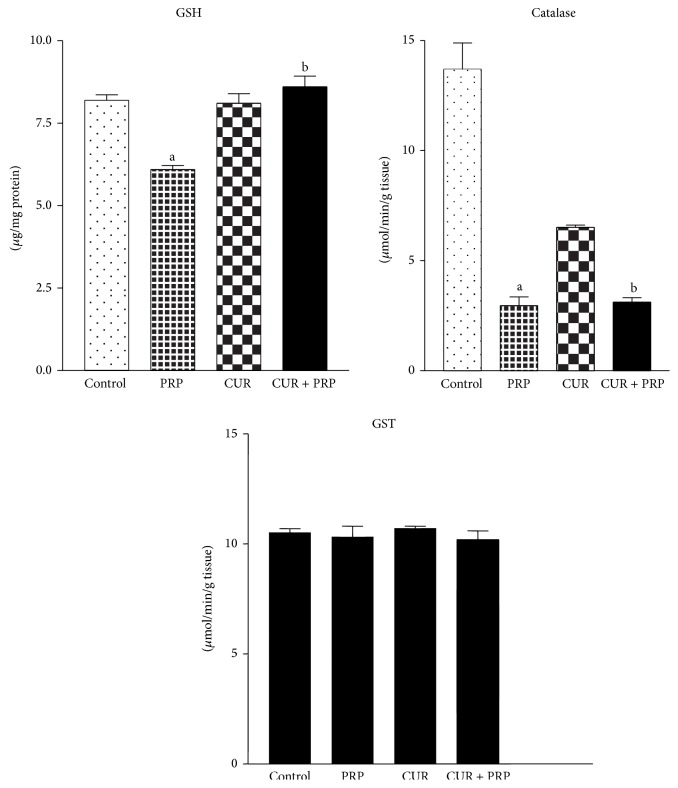
Effect of curcumin on reduced glutathione (GSH), catalase, and glutathione S-transferase (GST) in liver of rats orally treated with propanil. Values are expressed as mean ± SD for six animals per group. ^a^ *P* < 0.001 compared to control group. ^b^ *P* < 0.001 compared to curcumin group.

**Figure 3:**
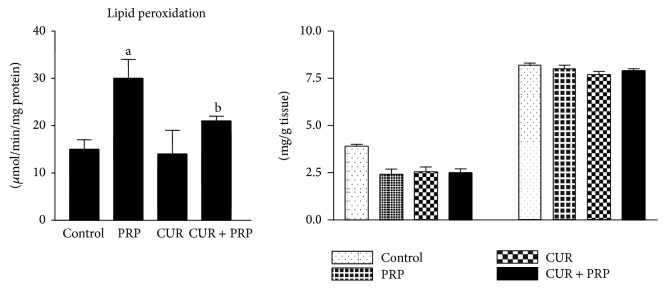
Effect of curcumin on lipid peroxidation, Vitamin C, and Vitamin E levels in liver of rats orally treated with propanil. Values are expressed as mean ± SD for six animals per group. ^a^ *P* < 0.001 compared to control group. ^b^ *P* < 0.001 compared to propanil group.

The ability of a cell or tissue to maintain its integrity is a function of the levels of the antioxidants present when compared with the level of the oxidants. The balance between these two determines the susceptibility of the cell or tissue to free radicals attack or oxidative stress. Results indicated that lipid peroxidation level was significantly (*P* < 0.001) increased by 103%, while GSH levels and catalase activity were significantly (*P* < 0.001) decreased in livers of rats treated with propanil (Figures 2 and 3) when compared with controls. However, there were no significant (*P* < 0.05) changes in the activity of GST and levels of Vit C and Vit E in liver of rats treated with propanil in comparison with the control group (Figure 3). Curcumin, administered alone, did not alter the lipid peroxidation and GSH levels when compared with the control. However, supplementation of curcumin to propanil-intoxicated rats significantly (*P* < 0.05) attenuated the increase in lipid peroxidation levels and restored the GSH levels to normal.

## 4. Discussion

The protective role of curcumin in propanil-induced toxicity in Wistar rats was examined in this study. Hematological and biochemical profiles of blood are known to provide important information about the internal environment of the organism. Curcumin, an antioxidant and anticarcinogenic substance, was reported to have a protective effect against liver damage and ferric nitrilotriacetate induced peroxidation of microsomal membrane lipids [28]. The protective action of curcumin against adverse effects of cisplatin had also been reported [29]. Curcumin could exert antioxidative effects either directly as a chemical antioxidant due to its ability to scavenge reactive oxygen and nitrogen free radicals or by modulating cellular defenses which themselves exert antioxidant effects [30, 31].

Due to its role in the transformation of environmental xenobiotics, the liver is at great risk of injury. Lipid peroxidation represents one of the most frequent reactions resulting from free radicals' attack on biological structures. In our study, the liver MDA level, index of lipid peroxidation, significantly increased in the propanil-treated rats. Supplementation with curcumin significantly attenuated the increase in lipid peroxidation levels. It is plausible that the mechanism of this protection could be the inhibition of reactive oxygen species generation by suppressing cytochrome P450 isozymes which are involved in the bioactivation of propanil to toxic reactive metabolites such as 3, 4-dichloroaniline [15, 32]. Reports are not available regarding Vitamins C and E concentrations in propanil-induced organ toxicity. A previous study had reported decrease in ascorbic acid levels in plasma and liver of rats treated with dimethylnitrosamine [33]. However, in the present investigation, a nonsignificant decrease in Vitamin C was observed in the liver of propanil-treated rats. This decrease may be indicative of increased oxidative stress, free radical formation, and simultaneous damage of the liver plasma membrane lipid bilayer arising from propanil intoxication. Reduction in Vitamin E content following treatment with 1, 2-dibromoethane had also been previously reported [34]. Data obtained for Vitamin E in liver of propanil exposed rats did not reveal any significant change in the vitamin content. Compared with the control, GSH concentrations in treated Wistar rats significantly decreased after exposure to propanil. This corroborated the findings that GSH concentrations in treated* Crucian carp* significantly decreased dose-dependently after acute exposure to dichloroaniline [35]. The depletion of GSH is suggestive of a deleterious effect of propanil on the antioxidant defense in the liver of rats. It is also consistent with the generation of oxidative stress, thereby reenforcing the role of GSH in molecular protective mechanisms that modulate cellular responses to toxic chemicals. The present study showed that administration of curcumin improved the GSH levels in rats. This finding is similar to the data reported by Piper et al. [36], who indicated an increase of the GSH level corresponding to curcumin dosage in rats fed curcumin, at doses up to 500 mg/kg body weight daily for 14 days.

GST detoxifies a variety of electrophilic compounds to less toxic forms by conjugation with–SH groups from GSH. In the present study, GST activity was not changed by any of the exposure schemes. This correlated with the findings that exposure of rats to cypermethrin in single and repeated doses had no relevant effect on GST activities [37]. In contrast, Saxena et al. [38] reported an increase in GST activity in rat red blood cells after exposure to chlorpyrifos and endosulfan. In addition, on the basis of our observations, a dose as low as 20 mg/kg propanil seemed not to induce such a level of oxidant stress and alteration of the antioxidant system in rats. In consonance with our earlier studies there was a significant decline in the activities of catalase after propanil administration [18], which may be due to oxidative stress; however, treatment with curcumin did not ameliorate this decline.

An increase in plasma cholesterol as a result of pesticide exposure may indicate loss of membrane integrity. Carlson and Kolmodin-Hedman [39] reported that the accumulation of pesticide in the liver was associated with the disturbance of lipid metabolism and elevation of serum cholesterol. In our study, cholesterol levels were mildly increased after the administration of propanil, but this was not statistically significant. Furthermore, there were no significant differences in the level of total cholesterol in propanil-treated rats when compared with the control. Although the curcumin + propanil group elicited a significant increase in the cholesterol levels when compared with control animals, however, there was no significant change when compared with the propanil-treated group. A plausible explanation for this observed effect may be due to synergetic prooxidant effects of propanil and curcumin combination. This is in agreement with a report that combining flavonoids with low level pesticides could modify their antioxidant potential and also trigger molecular events that could increase their health risks [40].

This study confirmed the liver damage in the propanil-treated group by the increase in AST and ALP levels in plasma. Exposure of propanil-intoxicated rats with curcumin normalized the activities of AST and ALP to their control values. The decrease in AST and ALP activities supports the hepatoprotective effects of curcumin, consistent with the findings that curcumin modulated the increased activity of marker enzymes and plasma lipid levels in nicotine-treated rats [41]. However, curcumin, when given alone, produced an increase in ALP activity, suggesting that the subacute administration of curcumin at the dose of 50 mg/kg used in this study may not be completely harmless. This agreed with an earlier report that curcumin, administered to human subjects at doses ranging from 0.9 to 3.6 g day^−1^ for 4 months, could cause some adverse effects including nausea, diarrhea, and increase in serum alkaline phosphatase and lactate dehydrogenase activities [42]. Clinical chemistry analysis of urea and creatinine could serve as prognostic indicators of renal dysfunction in the following exposure to toxicants. In the present study, we observed a nonsignificant increase in the level of creatinine in propanil-treated rats when compared to the control. This may possibly be due to increased catabolic state in the rats from prolonged appetite as a result of manifestation of acute oral toxicity [43]. However, coadminstration of curcumin did not mitigate the increased creatinine levels.

Hematological and biochemical profiles of blood can provide important information about the internal environment of the organism [44]. In this study, there were no prominent changes in the PCV and neutrophils. These findings are supported by the results of Garg et al. [45] who reported that treatment of broiler chicks with monocrotophos did not cause any significant change in erythrocyte count and PCV values. Furthermore, treatment of rats with curcumin + propanil increased total leukocyte count compared to rats in the control group. Our results are in accordance with the findings that treatment of rats with propetamphos plus propolis increased total leukocyte count. The increase observed in total leukocyte count might indicate an activation of the animal's immune system due to either stimulated lymphopoiesis or disturbance of the nonspecific immune system leading to the increased production of leukocytes [46, 47].

## 5. Conclusions

In summary, the present results suggested that curcumin protected against the liver toxicity induced by propanil treatment in rats. In this study, propanil elicited increase in AST and ALP activities. Furthermore, it had been suggested that lipid peroxidation might be a contributing factor to the development of liver toxicity. The significantly decreased activities of hepatic plasma markers (AST and ALP) and lipid peroxidation marker (malondialdehyde) along with normalizing of the endogenous GSH level suggest that curcumin is a strong antioxidant. We also observed that the curcumin + propanil combination resulted in a significant increase in cholesterol levels when compared with the control group suggesting a possible synergetic hyperlipidemic effect when curcumin combines with propanil.

## Figures and Tables

**Table 1:** Effect of curcumin (CUR) on some hematological parameters and total cholesterol values in rats treated with propanil (PRP).

Treatment	PCV, %	Total leucocyte, ×10^3^	Neutrophils	Total cholesterol (mg/dL)
CONTROL	41.5 ± 2.1	4.1 ± 0.4	60.3 ± 3.9	49 ± 2.8
PRP	40.8 ± 2.1	4.8 ± 0.9	51.2 ± 9.4	53 ± 4.1
CUR	41.8 ± 3.3	4.4 ± 0.4	55.0 ± 3.9	50.6 ± 4.4
CUR + PRP	41.0 ± 3.2	5.6 ± 0.2^a^	58.2 ± 5.1	55.2 ± 3.5

Values are mean ±SD of six rats in each group. ^a^ *P* < 0.001 compared to control group.
